# Cultivating Early Interest in Aging-Related Professions: Evaluation of a High School–Based “Silver Age Course”

**DOI:** 10.1093/geroni/igaf122.3910

**Published:** 2025-12-31

**Authors:** Xinxin Cai, Xinyu Yi, Xiang Li, Xue Bai

**Affiliations:** The Hong Kong Polytechnic University, Hong Kong, China (People’s Republic); The Hong Kong Polytechnic University, Hong Kong, China (People’s Republic); The Hong Kong Polytechnic University, Hong Kong, China (People’s Republic); The Hong Kong Polytechnic University, Hong Kong, China (People’s Republic)

## Abstract

**Background:**

The rising demand for care professionals highlights the need to cultivate early career interest. As high school students increasingly align studies with career goals, introducing aging-related careers presents a strategic opportunity. This study evaluated whether a weekly “Silver Age Course,” exposing students to aging, older adults and care professionals, could foster orientation toward care careers.

**Methods:**

A mixed-methods sequential explanatory design was adopted. Phase 1 involved a quasi-experiment with enrolled students (n = 28) and a control group (n = 31). Career orientation, willingness and confidence in communicating with older adults, understanding and application of knowledge and skills, impression of older adults, and compassion were measured before and after the course. Repeated-measures MANCOVA was used for analysis. Phase 2 included four rounds of reflective diaries (T1 to T4) from enrolled students (n = 28), analyzed using thematic trajectory analysis.

**Results:**

Significant time*group interactions were found for career orientation and understanding of knowledge and skills after controlling for social relationships (p < 0.05). Thematic trajectory analysis revealed that the course fostered care and aging-related career orientation, shifting students from non-care to care-related interests, vague to specific career goals, and unrelated aspirations to incorporating a caring mindset. These changes stemmed from students’ deeper understanding of aging, enhanced compassion, and maturity. Growth extended into school and family life, reinforcing motivation to care careers.

**Conclusions:**

High school students’ interest in aging-related professions can be fostered through experiential and project-based learning. Such programs support holistic growth of adolescents and help cultivate future care professionals.

